# Real-time magnetic resonance imaging guidance improves the yield of endomyocardial biopsy

**DOI:** 10.1186/1532-429X-18-S1-Q69

**Published:** 2016-01-27

**Authors:** Toby Rogers, Kanishka Ratnayaka, Parag Karmarkar, William Schenke, Jonathan R Mazal, Adrienne E Campbell-Washburn, Ozgur Kocaturk, Anthony Z Faranesh, Robert J Lederman

**Affiliations:** 1 National Heart Lung and Blood Institute, National Institues of Health, Bethesda, MD USA; 2 Department of Cardiology, Children's National Medical Center, Washington, DC USA; 3 Institute of Biomedical Engineering, Bogazici University, Istanbul, Turkey; 4 Russell H Morgan Department of Radiology and Radiological Sciences, Johns Hopkins University, Baltimore, MD USA

## Background

In current practice, the diagnostic yield of endomyocardial biopsy is low because the procedure is performed ‘blind' using X-ray fluoroscopy guidance and because many pathologies affect the myocardium in a patchy distribution. We hypothesized that biopsy performed under direct realtime MRI guidance would have superior diagnostic yield, in an animal model of focal myocardium pathology.

## Methods

An active visualization MR conditional bioptome was designed and built for transcatheter endomyocardial biopsy (MRI Interventions, Irvine, CA). A porcine model of focal myocardial pathology that enhances with late gadolinium enhancement imaging and that contained fluorescent tags that are easily identifiable under ultraviolet light was created. Under X-ray fluoroscopy, selective coronary artery catheterization was performed and 3 mL of fluorescent microspheres (NuFlow Hydrocoat, 15μm diameter, 5 million spheres/mL) was infused, followed by 2 mL of 100% ethanol to create a focal lesion. Animals were survived for min 7 days, after which each animal underwent both MRI and X-ray guided biopsy. Specimens were analysed using a Leica MZFIII dissecting microscope under transmitted or ultraviolet light with a 400-480 nm band pass filter.

## Results

**Figure 1:**
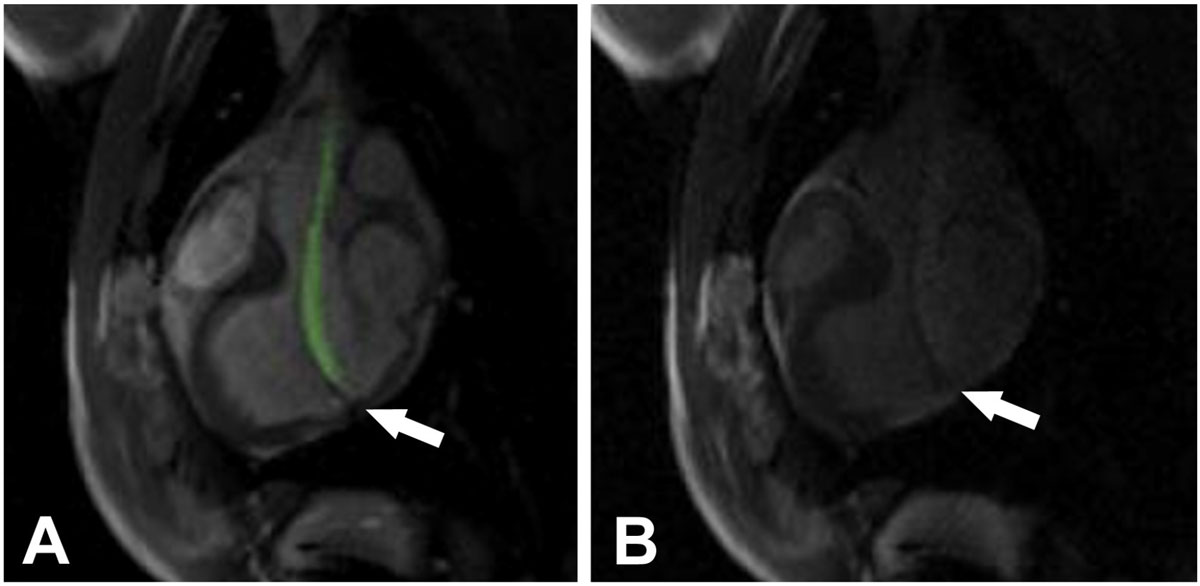
**Real-time MRI guided endomyocardial biopsy using an active visualization bioptome**.

**Figure 2:**
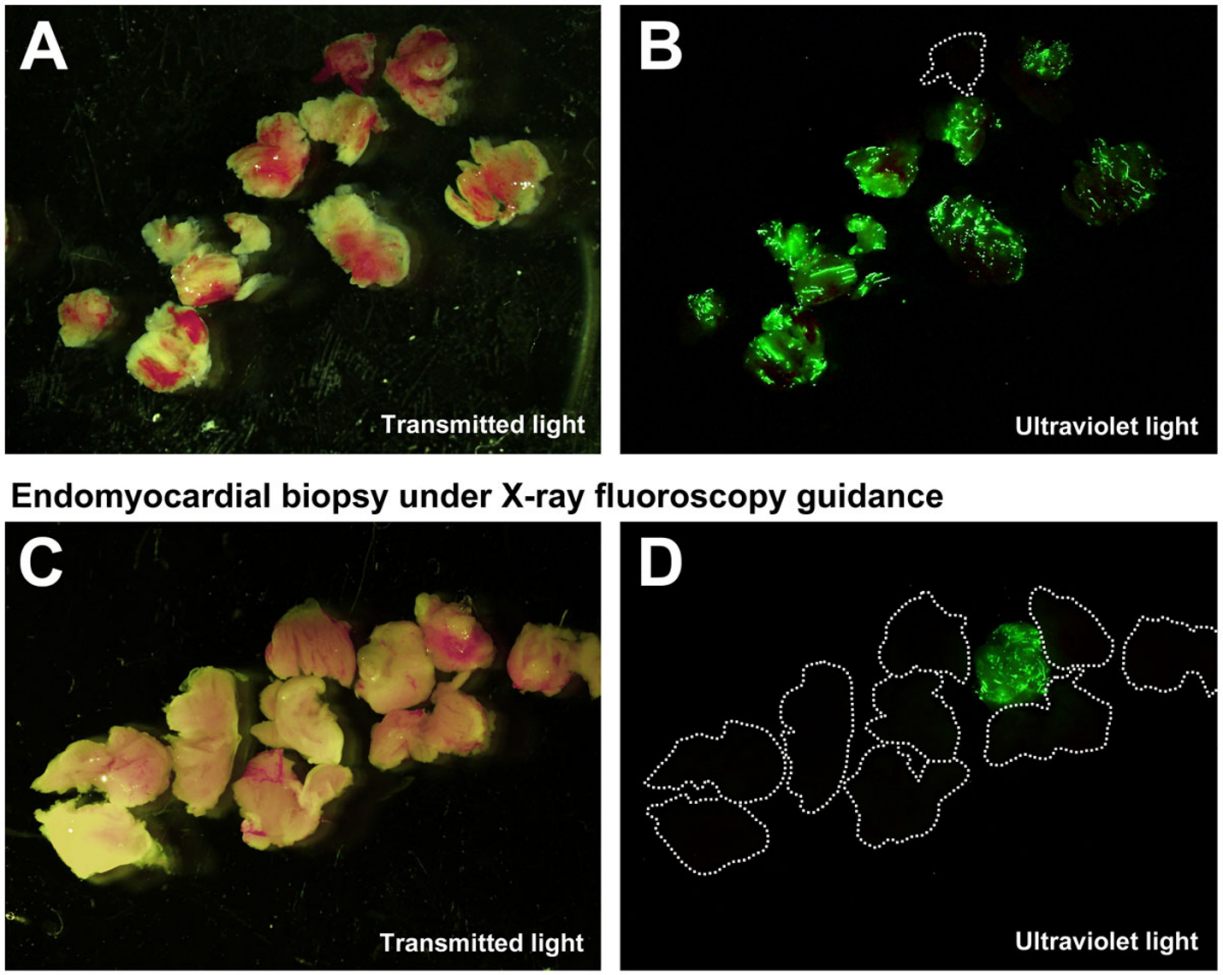
**Biopsy specimens under transmitted and ultraviolet light obtained using either real-time MRI or X-ray fluoroscopy guidance**.

The bioptome shaft was actively visualized under realtime MR imaging. The jaws appeared as a distinct signal void (arrow, Figure 1A). After administration of systemic gadolinium contrast, the lesion was visible using LGE or inversion recovery real-time MRI (Figure 1B). The bioptome was navigated to the pathology and specimens were collected. Animals were then transferred to an X-ray catheterization laboratory, where ‘conventional' fluoroscopy guided biopsy was performed. Examination of the biopsy specimens under ultraviolet light revealed fluorescent microspheres in 24/27 specimens obtained using MRI guidance compared with 7/28 specimens obtained using X-ray fluoroscopy guidance (Figure 2).

## Conclusions

Endomyocardial biopsy under direct real-time MRI guidance using an active visualization MRI-conditional bioptome is feasible. Using this bioptome we demonstrate targeted biopsy of focal myocardial pathology. Compared with X-ray fluoroscopy guided endomyocardial biopsy, MRI guidance substantially improves the diagnostic yield in an animal model of focal myocardial pathology.

